# The diabetes medication canagliflozin promotes mitochondrial remodelling of adipocyte via the AMPK-Sirt1-Pgc-1α signalling pathway

**DOI:** 10.1080/21623945.2020.1807850

**Published:** 2020-08-23

**Authors:** Xuping Yang, Qinhui Liu, Yanping Li, Qin Tang, Tong Wu, Lei Chen, Shiyun Pu, Yingnan Zhao, Guorong Zhang, Cuiyuan Huang, Jinhang Zhang, Zijing Zhang, Ya Huang, Min Zou, Xiongjie Shi, Wei Jiang, Rui Wang, Jinhan He

**Affiliations:** a Department of Pharmacy, State Key Laboratory of Biotherapy, West China Hospital, Sichuan University. Chengdu China; b Department of Pharmacy, The Affiliated Hospital of Southwest Medical University, Luzhou, China; c Laboratory of Clinical Pharmacy and Adverse Drug Reaction, State Key Laboratory of Biotherapy, West China Hospital, Sichuan University, Chengdu China; d College of Life Sciences, the Institute for Advanced Studies, Wuhan University, Wuhan, China; e Molecular Medicine Research Center, West China Hospital of Sichuan University, Chengdu, China; f Department of Cardiology, Yangpu Hospital, Tongji University, Shanghai, China

**Keywords:** Canagliflozin, adipocytes, mitochondrial biogenesis, energy homoeostasis, Pgc-1α

## Abstract

The diabetes medication canagliflozin (Cana) is a sodium glucose cotransporter 2 (SGLT2) inhibitor acting by increasing urinary glucose excretion and thus reducing hyperglycaemia. Cana treatment also reduces body weight. However, it remains unclear whether Cana could directly work on adipose tissue. In the present study, the pharmacological effects of Cana and the associated mechanism were investigated in adipocytes and mice. Stromal-vascular fractions (SVFs) were isolated from subcutaneous adipose tissue and differentiated into mature adipocytes. Our results show that Cana treatment directly increased cellular energy expenditure of adipocytes by inducing mitochondrial biogenesis independently of SGLT2 inhibition. Along with mitochondrial biogenesis, Cana also increased mitochondrial oxidative phosphorylation, fatty acid oxidation and thermogenesis. Mechanistically, Cana promoted mitochondrial biogenesis and function via an Adenosine monophosphate-activated protein kinase (AMPK) – silent information regulator 1 (Sirt1) – peroxisome proliferator-activated receptor γ coactivator-1α (Pgc-1α) signalling pathway. Consistently, *in vivo* study demonstrated that Cana increased AMPK phosphorylation and the expression of Sirt1 and Pgc-1α. The present study reveals a new therapeutic function for Cana in regulating energy homoeostasis.

**Abbreviations**: Ucp-1, uncoupling protein 1; cAMP, cyclic adenosine monophosphate; PKA, cAMP-dependent protein kinase A; SGLT, sodium glucose cotransporter; Cana, canagliflozin; T2DM: type 2 diabetes; Veh, vehicle; Pgc-1α, peroxisome proliferator-activated receptor γ coactivator-1α; SVFs, stromal-vascular fractions; FBS, bovine serum; Ad, adenovirus; mtDNA, mitochondrial DNA; COX2, cytochrome oxidase subunit 2; RT-PCR, real-time PCR; SDS-PAGE, sodium dodecyl sulphate-polyacrylamide gel electrophoresis; Prdm16, PR domain zinc finger protein 16; Cidea, cell death inducing DFFA-like effector A; Pgc-1β, peroxisome proliferator-activated receptor γ coactivator-1β; NRF1, nuclear respiratory factor 1; Tfam, mitochondrial transcription factor A; OXPHOS, oxidative phosphorylation; FAO, fatty acid oxidation; AMPK, Adenosine monophosphate-activated protein kinase; p-AMPK, phosphorylated AMPK; Sirt1, silent information regulator 1; mTOR, mammalian target of rapamycin; WAT, white adipose tissue; Fabp4, fatty acid binding protein 4; Lpl, lipoprotein lipase; Slc5a2, solute carrier family 5 member 2; ERRα, oestrogen related receptor α; Uqcrc2, ubiquinol-cytochrome c reductase core protein 2; Uqcrfs1, ubiquinol-cytochrome c reductase, Rieske iron-sulphur polypeptide 1; Cox4, cytochrome c oxidase subunit 4; Pparα, peroxisome proliferator activated receptor α; NAD^+^, nicotinamide adenine dinucleotide; Dio2, iodothyronine deiodinase 2; Tmem26, transmembrane protein 26; Hoxa9, homeobox A9; FCCP, carbonyl cyanide 4-(trifluoromethoxy) phenylhydrazone; Rot/AA, rotenone/antimycin A; OCR, oxygen consumption rate; Pparγ, peroxisome proliferator activated receptor γ; C/ebp, CCAAT/enhancer binding protein; LKB1, liver kinase B1; AUC, area under the cure; Vd, apparent volume of distribution.

## Introduction

Mitochondria have critical roles in many aspects of metabolism, and the cellular energy balance can be easily manipulated by affecting mitochondrial processes [1] such as oxidative phosphorylation and β-oxidation [2]. The utilization of glucose and fatty acid by cells depends to a large extent on the ability of mitochondria to metabolize these substrates and produce energy in the form of ATP and heat [3]. Any defect in mitochondrial function can result in the accumulation of lipids, which may affect various organs such as adipose tissue, the liver, and the skeletal muscle [4]. Peroxisome proliferator-activated receptor γ coactivator-1α (Pgc-1α) is a master transcriptional regulator of mitochondrial remodelling and biogenesis [5], and is tightly modulated by many transduction effectors such as adenosine monophosphate-activated protein kinase (AMPK), silent information regulator 1 (Sirt1) and mammalian target of rapamycin (mTOR) [6,7].

White adipose tissue (WAT) is a major endocrine organ that directly or indirectly affects many physiological functions [8]. Circulating cytokines secreted by fat tissue such as adiponectin [9] and asprosin [10] can regulate the cellular energy homoeostasis. Sufficient mitochondrial density or function are required to support the multiple biological processes of white adipocytes such as differentiation or remodelling. Any change in mitochondria function in adipocytes can impair the metabolism of this organism [11]. It has been reported that high levels of glucose and free fatty acids cause mitochondrial dysfunction in 3T3-L1 adipocytes [12], whereas another study revealed that the abundance of mitochondria and key genes related to mitochondrial function in WAT were significantly decreased in patients with metabolic disorders such as severe obesity and diabetes [13]. With this in mind, therapeutic interventions should be developed to improve oxidative capacity and energy homoeostasis in WAT.

SGLT2 inhibitors have emerged as a promising new class of glucose-lowering drugs for managing type 2 diabetes (T2DM), they increase urinary glucose excretion, thereby reducing hyperglycaemia [14]. Canagliflozin (Cana) is the first SGLT2 inhibitor approved by the US Food and Drug Administration [15]. In addition to improving blood glucose, Cana treatment also consistently reduces body weight in rodents [16] and patients with or without type 2 diabetes [17,18]. Weight loss associated with SGLT2 inhibitors was generally considered to result from steady caloric loss via increased glucosuria [19], accompanied by a chain of metabolic response [20,21]. However, Cana was also reported to target liver [22] and cancer [23] independent of SGLT2 inhibition. To date, no study has assessed the impact of Cana on adipocyte *in vitro*. Therefore, it is still unclear whether Cana directly works on adipose tissue.

In this study, we have uncovered a critical role of Cana in directly regulating mitochondrial biogenesis, oxidative metabolism and thermogenic activity in adipocyte, and finally increasing cellular energy metabolism. Mechanistically, Cana may induce Pgc-1α expression via an AMPK-Sirt1 signalling pathway.

## Methods

### Animals

C57BL/6 J male mice (8 weeks old; n = 6) were treated with Cana (Meilunbio®, Dalian, China) or equal amounts of starch (as vehicle group) for 8 weeks (0.03% w/w Cana was mixed with powdered chow diet). On the basis of daily food intake and body weight, 0.03% (w/w) was approximately equivalent to 20 ~ 30 mg/kg [16]. This dosage is known to inhibit SGLTs efficiently [16]. All mice were maintained in ventilated cages with a 12 h light/dark cycle and free access to food and water. CO_2_ inhalation euthanasia was used to euthanize the mice. Kidney and subcutaneous adipose tissue were dissected and divided into portions for mRNA or protein analysis. All animal procedures were performed in accordance with the Animal Care and Use Committee of Sichuan University.

### Isolation and Differentiation of primary adipocytes

Stromal-vascular fractions (SVFs) were isolated from subcutaneous adipose tissue as described [24]. In brief, subcutaneous adipose tissue was dissected from 4- to 6-week-old C57BL/6 J mice, then digested for 30 min at 37°C in isolation buffer (containing 1 mg/ml collagenase, 0.123 M NaCl, 5 mM KCl, 1.3 mM CaCl_2_, 5 mM glucose, 100 mM HEPES, and 4% BSA), and centrifuged at 700 g for 10 min at room temperature. Cell pellets were washed twice and resuspended in DMEM/F12 medium containing 10% foetal bovine serum (FBS) (Gibco, NY, USA). For differentiation, cells at 100% confluence (Day 0) were exposed to induction medium containing 10% FBS, 0.5 mM isobutylmethylxanthine (Sigma-Aldrich, MO, USA), 1 μM dexamethasone (Sigma-Aldrich, MO, USA), 1 μM rosiglitazone (Meilunbio®, Dalian, China), 1 nM T3 (Sigma-Aldrich, MO, USA) and 10 μg/ml insulin (Sigma-Aldrich, MO, USA). Two days after induction (from Day 3), cells were switched to maintenance medium containing 10% FBS, 1 nM T3, 10 μg/ml insulin until they were ready for harvesting (generally days 6–7 post differentiation). Differentiated primary adipocytes were treated with 10 μM Cana (Meilunbio®, Dalian, China) or drug vehicle (DMSO, Sigma-Aldrich, MO, USA) for 48 h and collected for further analysis. All group data subjected to statistical analysis were repeated in at least three independent experiments, each in duplicate or triplicate.

### Mitochondrial labelling and immunofluorescence

Differentiated primary adipocytes were treated with 10 μM Cana or drug vehicle (DMSO) for 48 h and collected for further analysis. For mitochondrial labelling, differentiated adipocytes were treated with 10 μM Cana or vehicle for 48 h and labelled with a mitochondrial probe. Following protocols recommended by the manufacturer, Mito Tracker Red CMXRos (200 nM) (Thermo Fisher Scientific, MA, USA) was used to stain mitochondria for 30 min at 37°C, then cell nucleus was stained with DAPI (Meilunbio®, Dalian, China). The fluorescence images were taken by confocal laser scanning microscopy (CLSM) (Leica, Wetzlar, Germany). The red fluorescence intensity quantification was detected (excitation wavelength of 579 nm and emission wavelength at 599 nm) using a Bio-Tek fluorescent microplate reader (BioTek Instruments, Inc.). For immunofluorescence, TOMM20 primary antibody was used at a dilution of 1:200 and incubated overnight at 4°C, then adipocytes were incubated with secondary anti-rabbit Alexa Fluor 488 antibody for 1 h at room temperature, and incubated with DAPI dye for 2 min. The fluorescence images were taken by confocal laser scanning microscopy. All representative images were repeated in at least three independent experiments.

### Adenoviral infection

For adenoviral infection, differentiated adipocytes were infected with control or (Ad)-shPgc-1α adenovirus (10 μl of 2 × 10^10^ Pfu) for 48 h, and then treated with 10 μM Cana or vehicle for 48 h. All data obtained from cell samples were repeated in at least three independent experiments, each in triplicate.

### Real-time PCR analysis

Total RNA of tissue and adipocytes were extracted with Trizol (Invitrogen, CA, USA). Reverse transcription of total RNA (1 μg) was performed with a high-capacity cDNA reverse transcription kit (Takara, Shiga, Japan). Amplification reactions were conducted using a SYBR Green qRT-PCR Kit (Bio-Rad Laboratories, CA, USA) with the Bio-RAD CFX 96 PCR system [25]. The amplification of each gene was verified by melting points. All primer sequences are listed in Table S1.

### Mitochondrial DNA measurement

Total DNA of adipocyte was extracted by conventional phenol-chloroform method as described [24]. DNA concentration was assessed with a Nanodrop 2000 (Thermo Fisher Scientific, MA, USA). Mitochondria DNA (mtDNA) was amplified by using primers specific for the mitochondrial cytochrome oxidase subunit 2 (Cox 2), a marker of mitochondrial copy number [26], then normalized to genomic DNA coded by 18s. Primer sequences for Cox 2 and 18s were as follows: COX 2: forward 5ʹ-ATAACCGAGTCGTTCTGCCAAT-3ʹ and reverse 5ʹ-TTTCAGAGCATTGGCCATAGAA-3ʹ; and 18s: forward 5ʹ- TGTGTTAGGGGACTGGT GGACA −3ʹ and reverse 5ʹ- CATCACCCACTTACCCCCAAAA-3ʹ. mtDNA copy number relative to genomic DNA content was quantitatively analysed with the Bio-RAD CFX 96 PCR system.

### Oxygen consumption rate measurement

Primary fat SVF cells from subcutaneous adipose tissue were plated in an XFp-well microplate (Seahorse Bioscience, MA, USA), and differentiated for 8 d and treated with vehicle (DMSO) or 10 μM Cana for 48 h. Oxygen consumption rate (OCR) was measured at 37°C by using an XFp analyser (Seahorse Bioscience, MA, USA) in accordance with the manufacturer’s instructions. In total, 1 μM oligomycin (Seahorse Bioscience, MA, USA), 0.5 μM carbonyl cyanide 4-(trifluoromethoxy) phenylhydrazone (FCCP) (Seahorse Bioscience, MA, USA) and 0.5 μM rotenone/antimycin A (Rot/AA) (Seahorse Bioscience, MA, USA) were delivered to detect basal respiration, uncoupled respiration, maximal respiration and non-mitochondrial respiration, respectively. Relative OCR is calculated by subtracting OCR measured after antimycin addition from basal OCR, from OCR after oligomycin addition, or from OCR after FCCP addition.

### Western blotting analysis

Western blotting analyses of tissue and adipocytes were performed as described [25]. Total protein was extracted from cells using RIPA buffer (Sigma-Aldrich, MO, USA), protease inhibitor cocktail and phosphatase inhibitor cocktail (Thermo Fisher Scientific, MA, USA). Protein concentration was measured using a commercially available assay (Thermo Fisher Scientific, MA, USA) according to the manufacturer’s protocol. Extracted proteins were separated by sodium dodecyl sulphate-polyacrylamide gel electrophoresis (SDS-PAGE) and then transferred to polyvinylidene fluoride membranes (Millipore, MA, USA). Primary antibodies for western blotting analysis are listed in Table S2. All immunoreactive bands were visualized and analysed by densitometric scanning and Image J software. All representative images were repeated in at least three independent experiments.

### Statistical analysis

Student *t*-test was used for comparing two groups. One-way ANOVA followed by the Dunnett post hoc test was used for multiple comparisons versus the control group (GraphPad Software). *p* < 0.05 was considered statistically significant. Data are shown as mean ± SEM.

## Results

### Canagliflozin promotes mitochondrial biogenesis

**Figure 1.**
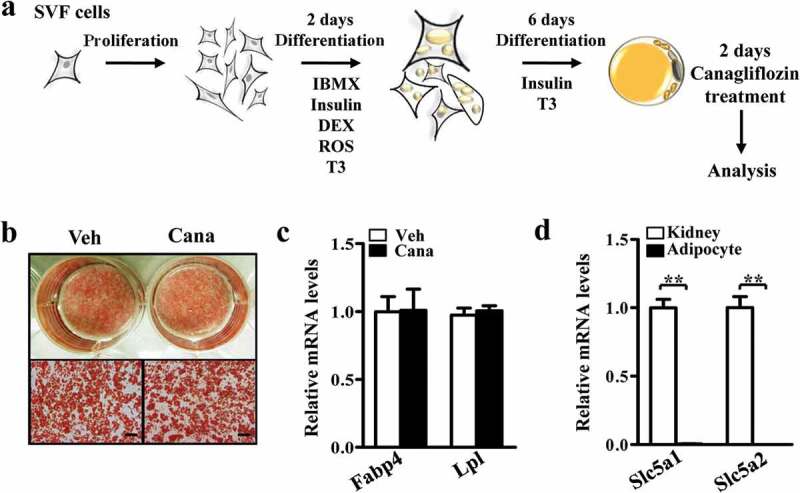
Isolation and differentiation of primary adipocyte. Differentiated primary adipocytes were treated with Canagliflozin (Cana) 10 μM or vehicle for 48 h. (a) Flow chart of isolation and differentiation of primary adipocyte. (b) Oil red staining of differentiated adipocytes. Scale bar, 50 μm. All representative images were repeated in at least three independent experiments. (c) Fabp4 and Lpl mRNA levels in differentiated adipocytes. (d) Slc5a1 and Slc5a2 mRNA levels in kidney from C57BL/6 J male mice and differentiated adipocytes. All group data subjected to statistical analysis were repeated in at least three independent experiments, each in duplicate or triplicate. Data are presented as mean ± SEM and *p < 0.05, **p < 0.01 compared to control group. IBMX, isobutyl-methylxanthine, DEX, dexamethasone; ROS, rosiglitazone

To determine the direct effect of Cana on adipose tissue, we isolated mesenchymal stem cells from subcutaneous adipose tissue of C57BL/6 J mice, differentiated into mature adipocyte, and then treated with Cana or DMSO for 48 h (Figure 1(a)). The concentration of Cana was justified according to the peak plasma concentrations achieved following therapeutic doses in humans [27]. Oil red O staining results showed that more than 90% of cells were differentiated in both groups (Figure 1(b)). No significant differences were observed in the levels of fatty acid binding protein 4 (Fabp4) and lipoprotein lipase (Lpl), two fat cell-specific markers, between Cana-treated and vehicle groups (Figure 1(c)). Given the previously reported role of SGLT2 inhibitors on kidney, we next examined the expression of Solute carrier family 5 member 1 and 2 (Slc5a1/2, gene encoding SGLT1 and SGLT2). RT-PCR analysis revealed that Slc5a1 and Slc5a2 were highly expressed in the kidney and barely detectable in adipose tissue (Figure 1(d)).

**Figure 2.**
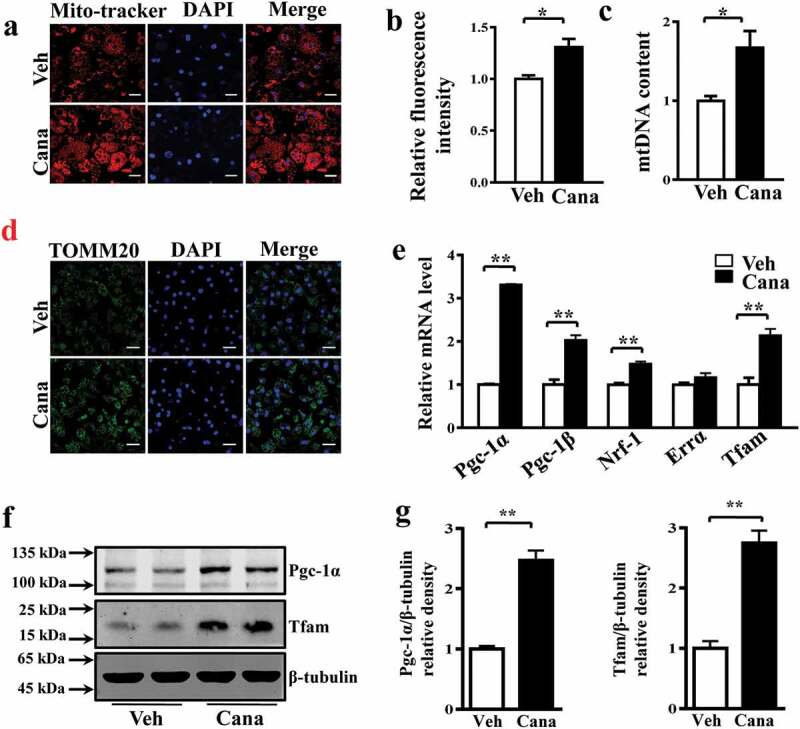
Cana directly promotes mitochondrial biogenesis. Differentiated primary adipocytes were treated with Canagliflozin (Cana) 10 μM or vehicle for 48 h. (a) Mitochondrial staining of differentiated adipocytes. Scale bar, 10 μm. (b) Relative fluorescence intensity of Mitochondrial staining in (a). (c) Relative mtDNA content of differentiated adipocytes. (d) Representative images of TOMM20 immunofluorescence (in green) on differentiated adipocytes. Nuclei were stained with DAPI (in blue). Scale bars, 25 μm. (e) mRNA levels of gene involved in mitochondrial biogenesis in differentiated adipocytes. (f, g) Protein levels of Pgc-1α and Tfam in differentiated adipocytes. The relative average protein level was determined by densitometry and normalized with β-tubulin. All group data subjected to statistical analysis were repeated in at least three independent experiments, each in duplicate or triplicate. Data are presented as mean ± SEM and *p < 0.05, **p < 0.01 compared to control group

Then, we used a Mito-Tracker to label mitochondria in adipocytes, and the results indicated that the fluorescence intensity in Cana-treated group was higher than the control group (Figure 2(a-b)). We further analysed the mitochondria content of primary adipocytes via determination of copy number and immunofluorescent intensity. The results confirmed Cana can increase the mitochondrial biogenesis (Figure 2(c–d)). Mitochondrial transcription factor A (Tfam, encoded by the nuclear genome) is a master transcription factor to regulate the transcription of the mitochondrial genome and regulated by the transcription factors NRF (nuclear respiratory factor)-1/2 [8,28]. NRFs are under the control of coactivators of mitochondrial gene transcription including Pgc-1α and Pgc-1β [7]. RT-PCR analysis showed that Cana treatment significantly increased the expression of Pgc-1α and Pgc-1β, and their target genes Nrf-1 and Tfam in primary subcutaneous adipocytes (Figure 2(e)). Changes in the levels of Pgc-1α and Tfam were also confirmed by western blotting analysis (Figure 2(f–g)). Taken together, these results indicate that Cana can induce mitochondrial biogenesis in adipocytes.

### Canagliflozin promotes mitochondrial OXPHOS and FAO

**Figure 3.**
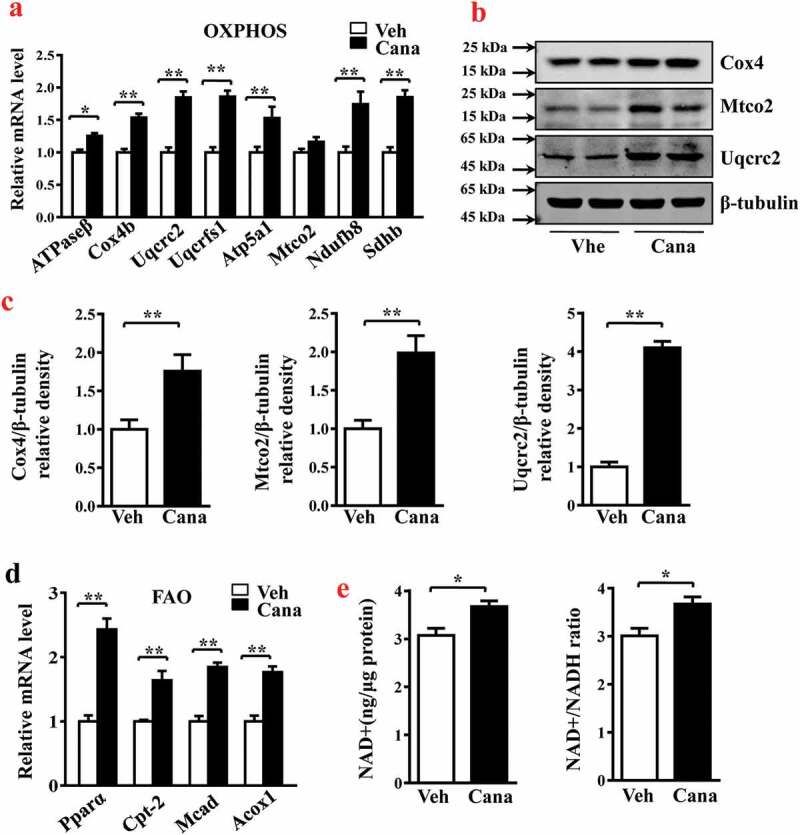
Cana directly promotes mitochondrial OXPHOS and FAO. Differentiated primary adipocytes were treated with Canagliflozin (Cana) 10 μM or vehicle for 48 h. (a) mRNA levels of gene involved in mitochondrial OXPHOS in differentiated adipocytes. (b-c) Protein levels of Cox4, Mtco2 and Uqcrc2 in differentiated adipocytes. The relative average protein level was determined by densitometry and normalized with β-tubulin. (d) mRNA levels of gene involved in mitochondrial FAO in differentiated adipocytes. (e) Intracellular NAD^+^ level and NAD^+^/NADH ratio in differentiated adipocytes. All group data subjected to statistical analysis were repeated in at least three independent experiments, each in duplicate or triplicate. Data are presented as mean ± SEM and *p < 0.05, **p < 0.01 compared to control group

Oxidative phosphorylation (OXPHOS) of metabolic substrates and fatty acids oxidation (FAO) are two critical biological processes of mitochondria in adipocytes [29]. Several studies have reported an association between insulin resistance and impaired mitochondrial oxidative function [8,30]. Considering the effects of Cana on mitochondrial biogenesis, we examined whether the increased number of mitochondria in adipocytes could translate to enhanced oxidative capacity. As shown in Figure 3, this compound significantly increased the mRNA and protein levels of mitochondrial enzymes involved in the respiratory chain, including cytochrome c oxidase subunit 4 (Cox4) and ubiquinol-cytochrome c reductase core protein 2 (Uqcrc2) (Figure 3(a–c)). In adipocytes, the enhanced activity of mitochondria can increase lipid utilization. Our results indicated that Cana significantly increased the mRNA levels of genes mediating fatty acid metabolism such as peroxisome proliferator activated receptor α (Pparα) and medium-chain acyl-CoA dehydrogenase (Mcad) (Figure 3(d)). In addition, the Cana-induced increase in mitochondrial function was supported by the accumulation of nicotinamide adenine dinucleotide (NAD+) (Figure 3(e)). These findings indicate that Cana can promote oxidative phosphorylation and fatty acid oxidation in mitochondria.

### Canagliflozin promotes thermogenesis and energy metabolism

**Figure 4.**
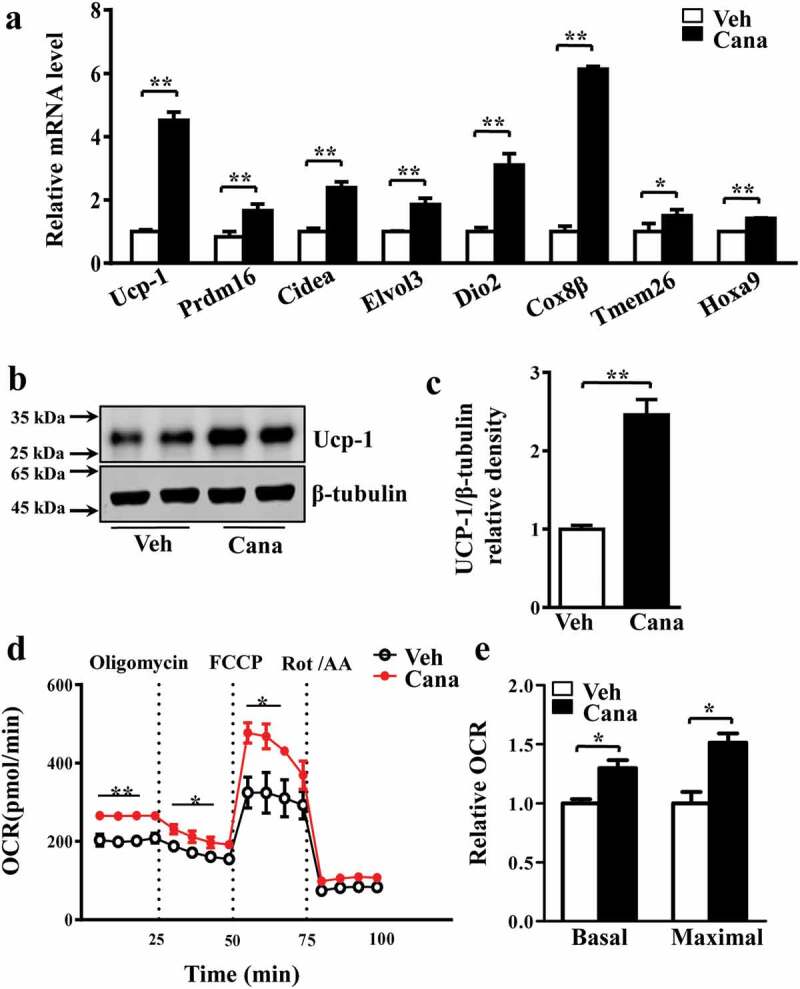
Cana directly promotes thermogenesis and energy metabolism. Differentiated primary adipocytes were treated with Canagliflozin (Cana) 10 μM or vehicle for 48 h. (a) mRNA levels of gene involved in thermogenesis in differentiated adipocytes. (b-c) Protein levels of Ucp-1 in differentiated adipocytes. The relative average protein level was determined by densitometry and normalized with β-tubulin. (d) Oxygen consumption rate (OCR) in differentiated adipocytes in basal conditions, in the presence of 1 μM oligomycin, 0.5 μM FCCP, or 0.5 μM rotenone/antimycin A. (e) Relative OCR in (d). All group data subjected to statistical analysis were repeated in at least three independent experiments, each in duplicate or triplicate. Data are presented as mean ± SEM and *p < 0.05, **p < 0.01 compared to control group

It has been reported that activation of brown fat and white fat browning enhances thermogenesis, which can improve some metabolic disorders such as obesity and diabetes [31], and these processes associated with increased mitochondrial function [32,33]. This prompted us to investigate the effects of Cana on thermogenesis. Results of RT-PCR and western blotting analyses revealed that the level of Ucp-1, a mitochondrial protein responsible for thermogenic respiration, was significantly up-regulated (Figure 4(a–c)). Other thermogenic genes like Cell death-inducing DFFA-like effector A (Cidea), iodothyronine deiodinase 2 (Dio2) and Cytochrome c oxidase subunit 8β (Cox8β), or beige-selected genes inducing transmembrane protein 26 (Tmem26) and homeobox A9 (Hoxa9) were also increased by Cana treatment (Figure 4(a)). High metabolic rate of adipocyte depends on multiple biological processes including OXPHOS, FAO and thermogenesis, accompanied by the increased oxygen consumption [8,34,35]. We then investigated whether Cana could alter the cellular oxygen consumption measured by using an Agilent Seahorse XFp Analyser. Indeed, Cana significantly increased the basal and maximal respiratory capacity (Figure 4(d–e)), thereby confirming the effect of Cana in the regulation of oxidative respiration and energy expenditure.

### Pgc-1α knockdown abolishes the effects of Cana on primary adipocyte

**Figure 5.**
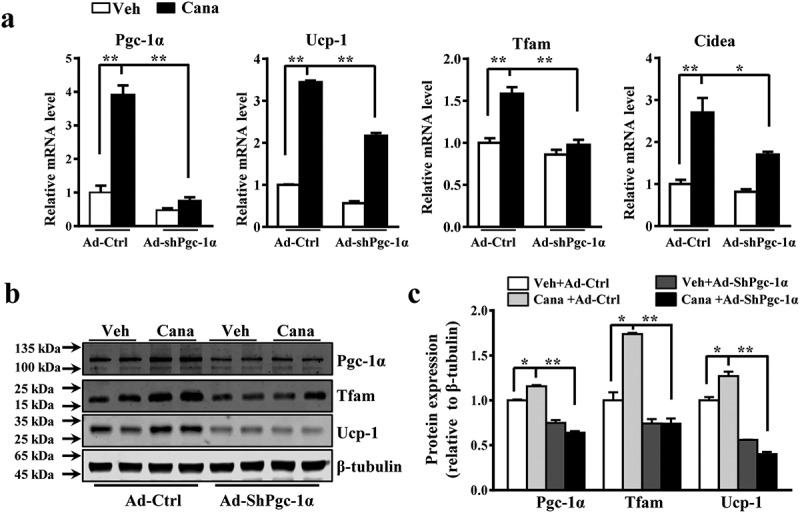
Pgc-1a knockdown abolishes the effects of Cana on primary adipocyte. Differentiated primary adipocytes were treated with Canagliflozin (Cana) 10 μM or vehicle for 48 h. (a) mRNA levels of Pgc-1α, Ucp-1, Tfam and Cidea in control and in Pgc-1α knockdown (Ad-shPgc-1α) differentiated primary adipocyte. (b-c) Protein levels of Pgc-1α, Ucp-1 and Tfam in control and in Pgc-1α knockdown (Ad-shPgc-1α) differentiated primary adipocyte. The relative average protein level was determined by densitometry and normalized with β-tubulin. All group data subjected to statistical analysis were repeated in at least three independent experiments, each in duplicate or triplicate. Data are presented as mean ± SEM and *p < 0.05, **p < 0.01 compared to control group

Based on the observation that Cana promoted adipocyte mitochondrial biogenesis, thermogenic activity and oxygen consumption *in vitro*, we next explore the specific mechanism of Cana in these biological processes. Given the essential role of Pgc-1α in white fat browning and mitochondrial function, we hypothesized that the effects of Cana on primary adipocytes might be mediated by Pgc-1α. A shPgc-1α adenovirus was used to ablated the expression of Pgc-1α in primary adipocytes. We found Cana could significantly increase the mRNA or protein levels of Pgc-1α and its target genes in the control group (Figure 5(a–c)). However, these effects were largely abolished when Pgc-1α knockdown (Figure 5(a–c)). These results confirmed that Pgc-1α was necessary for Cana’ metabolic action in adipocytes.

**Figure 6.**
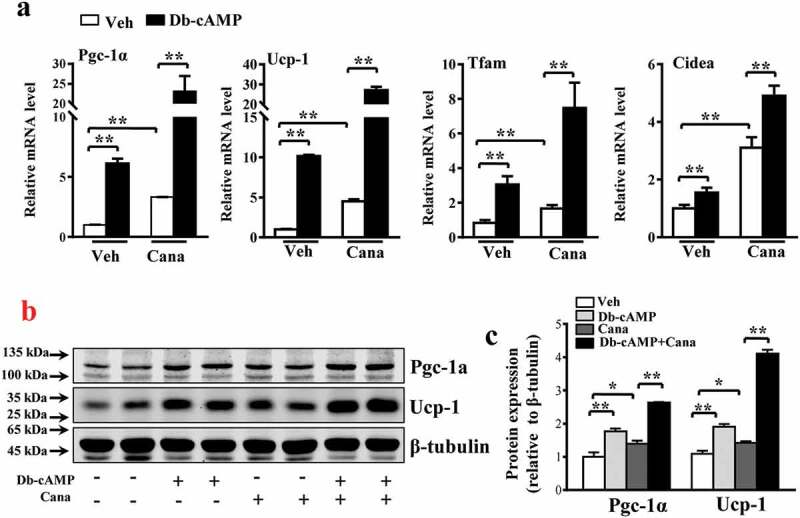
Cana enlarges the effects of Db-cAMP on differentiated primary adipocyte. Differentiated primary adipocyte preincubated with Cana 10 μM or vehicle for 48 h, then stimulated with Db-cAMP 10 μM for 4 h. (a) mRNA levels of Pgc-1α, Ucp-1, Tfam and Cidea. (b-c) Protein levels of Pgc-1α and Ucp-1. The relative average protein level was determined by densitometry and normalized with β-tubulin. All group data subjected to statistical analysis were repeated in at least three independent experiments, each in duplicate or triplicate. Data are presented as mean ± SEM and *p < 0.05, **p < 0.01 compared to control group

Db-cAMP, a cAMP analogue, has been reported to activate the PKA pathway and up-regulate Pgc-1α expression [36]. The levels of Pgc-1α and its downstream genes were increased by Db-cAMP treatment and further increased after Cana treatment (Figure 6(a)). Cana increased Pgc-1α expression by approximately 7-fold and Ucp-1 expression by approximately 10-fold (Figure 6(a)), consistent with the results of western blotting analysis (Figure 6(b–c)). These findings indicate that Pgc-1α is a regulator of mitochondrial biogenesis and thermogenesis.

### Cana promotes AMPK activation and induces Sirt1 expression

**Figure 7.**
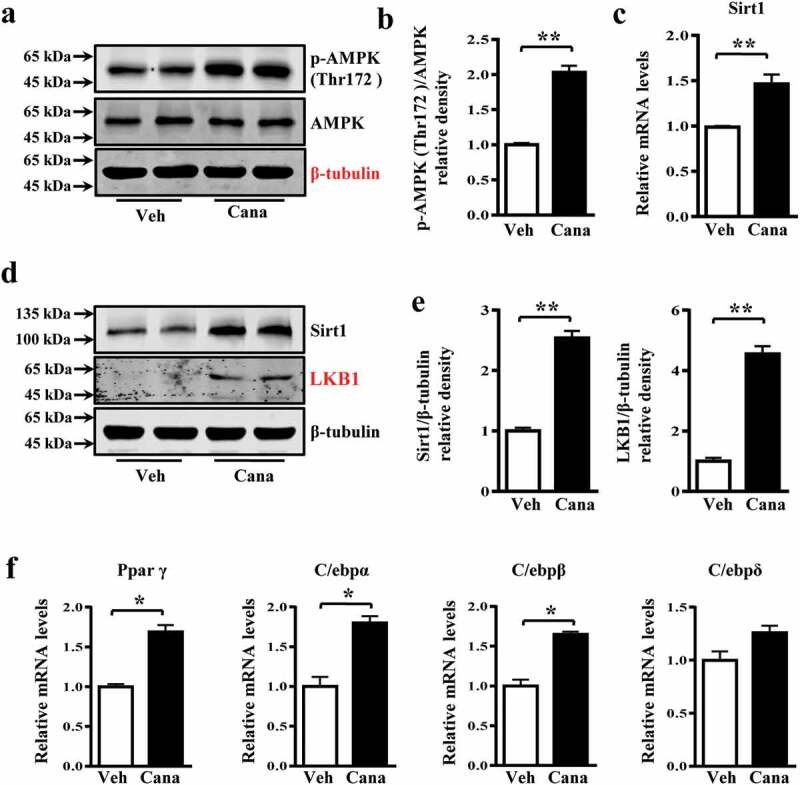
Cana induces Sirt1 expression and promotes AMPK activation. Differentiated primary adipocytes were treated with Canagliflozin (Cana) 10 μM or vehicle for 48 h. (a-b) Protein levels of AMPK and phosphorylation levels of AMPK (p-AMPK) at Thr 172 site in differentiated adipocytes. The relative average phosphorylation levels of AMPK determined by densitometry and normalized with AMPK. (c) mRNA levels of Sirt1 in differentiated adipocytes. (d-e) Protein levels of Sirt1 and LKB1 in differentiated adipocytes. The relative average protein level was determined by densitometry and normalized with β-tubulin. (f) mRNA levels of Ppar γ, C/ebpα, C/ebpβ and C/ebpδ in differentiated adipocytes. All group data subjected to statistical analysis were repeated in at least three independent experiments, each in duplicate or triplicate. Data are presented as mean ± SEM and *p < 0.05, **p < 0.01 compared to control group

**Figure 8.**
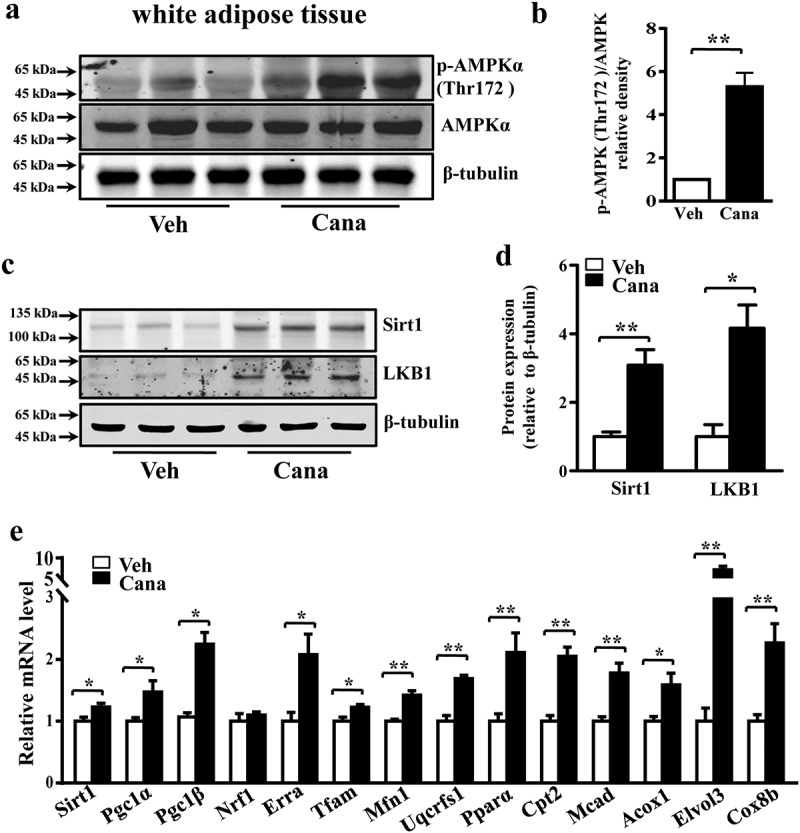
Cana promotes AMPK activation *in vivo*. Mice were treated with Canagliflozin (Cana) or vehicle for 8 weeks. (a-b) Protein levels of AMPK and phosphorylation levels of AMPK (p-AMPK) at Thr172 site in subcutaneous adipose tissue. The relative average phosphorylation levels of AMPK determined by densitometry and normalized with AMPK. (c-d) Protein levels of Sirt1 and LKB1 in differentiated adipocytes. The relative average protein level was determined by densitometry and normalized with β-tubulin. (e) mRNA levels of gene involved in mitochondrial remodelling of subcutaneous adipose tissue (n = 6). All group data subjected to statistical analysis were repeated in at least three independent experiments. Data are presented as mean ± SEM and *p < 0.05, **p < 0.01 compared to control group

AMPK and Sirt1 are important energy regulatory factors, and both can stimulate mitochondrial biogenesis and regulate glucose homoeostasis [5,37]. AMPK phosphorylation or Sirt1 deacetylation have been reported to directly activate Pgc-1α, thereby regulating mitochondrial function [28]. Indeed, Cana treatment increased the levels of phosphorylated AMPK, and the expression of Sirt1 and liver kinase B1 (LKB1), a kinase upstream of AMPK, in adipocytes (Figure 7(a–e)). Moreover, the expression of Sirt1-downstream nuclear transcriptional regulators such as peroxisome proliferator-activated receptor γ (Pparγ), CCAAT/enhancer-binding protein (C/ebp)-α and-β were also up-regulated (Figure 7(f)), which have been reported to regulate the activity of Pgc-1α [29]. Therefore, the effects of Cana on mitochondrial function might be, at least partly, mediated by an AMPK/Sirt1/Pgc-1α signalling pathway. To determine whether Cana can activate AMPK *in vivo*, mice were treated with Cana by oral gavage for 8 weeks. Cana treatment was found to up-regulate the levels of phosphorylated AMPK, and the expression of Sirt1 and LKB1 (Figure 8(a–d)), and promote mitochondrial remodelling (Figure 8(e)) in white adipose tissue.

**Figure:**
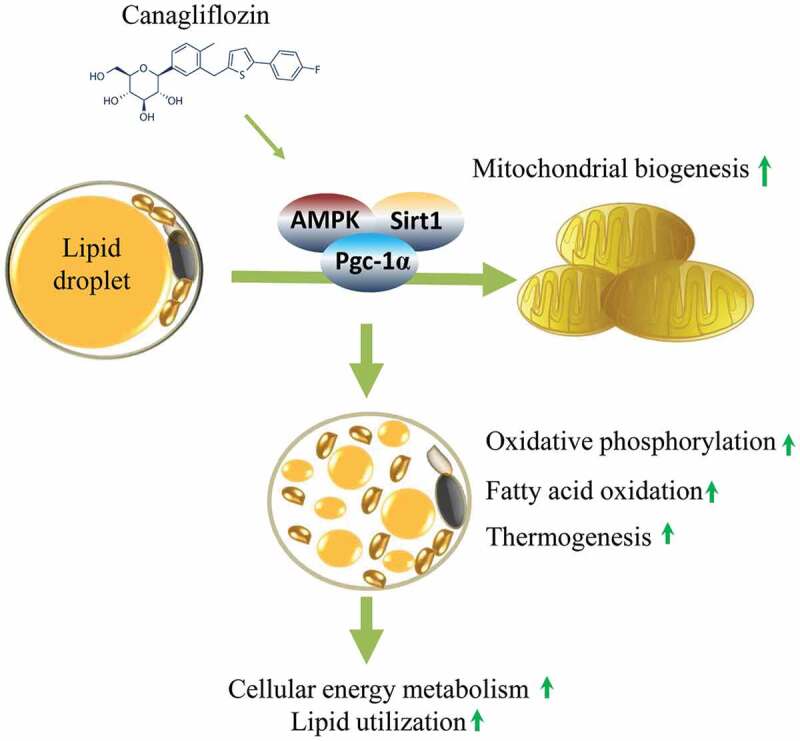

## Discussion

Over the past years, the diabetes medication Cana has been characterized as an anti-hyperglycaemic agent by inhibiting renal glucose reabsorption [19]. Beyond their well-characterized role in glucose regulation, this agent provided durable weight loss in several clinical trials [14,17,38]. When compared with other SGLT2 inhibitors, Cana resulted in the greatest reduction in body weight in one meta-analysis [39]. Further research indicates that roughly two-thirds of the reduction in body weight was from fat mass in Cana-treated group [17]. In addition, chronic Cana treatment for 8 weeks protected mice from high-fat-diet-induced obesity, inflammation and steatohepatitis [16]. However, these effects were considered to be associated with metabolic response induced by glucosuria [14]. By performing *in vitro* environment, we found that clinically achievable concentrations of Cana could directly promote mitochondrial biogenesis, oxidative metabolism and thermogenic activity of differentiated primary adipocyte independently of SGLT2 inhibition, and finally increase cellular energy metabolism.

At a cellular level, adaptive thermogenesis consists of several independent processes: mitochondrial biogenesis and upregulating mitochondrial enzymes involved in the respiratory chain and specific uncoupling proteins such as Ucp-1 [40]. In addition, studies have demonstrated that mitochondrial homoeostasis plays a key role in morphological and molecular characteristic maintenance of adipocyte browning [41,42]. A decrease in mitochondrial number is tightly coupled with the beige-to-white adipocyte transition [41]. Previous researches have demonstrated that SGLT2 inhibition results in a progressive shift to enhanced usage of fat [43,44]. Meanwhile, SGLT2 inhibition by Empagliflozin promotes fat utilization and browning of white adipose tissue in diet-induced obese mice [21]. We cannot rule out that SGLT2 inhibition induced by Cana in kidney may also participate to the shift from carbohydrate to lipid. Whatever, the increased mitochondrial function and thermogenesis of adipose tissue should, at least partially, contribute to the higher energy metabolism *in vivo*.

In this study, we treated mice with Cana for 8 weeks and found this compound promoted AMPK phosphorylation and mitochondrial remodelling of white adipose tissue. In contrast, Hawley et al. indicated that Cana promotes AMPK activation in mouse liver, but not in muscle, adipose tissue, or spleen [22]. We reasoned that this discrepancy may be due to the differences in treatment courses. In Hawley et al.’s study, mice were administered by oral gavage (100 mg/kg), and 3 h later tissues were collected [22]. Therefore, it’ s an acute treatment. In our study, mice were treated for 8 weeks. In Devineni et al.’s study, a single- and multiple-dose administration of Cana showed significant differences in pharmacokinetic parameters including area under the curve (AUC) and apparent volume of distribution (Vd) [45]. Therefore, we speculate that the distribution of Cana is not stable enough in 3 h’s acute treatment.

AMPK is an energy sensor that maintains cellular energy homoeostasis, and AMPK activation participates in multiple biological events including mitochondrial biogenesis and oxidative function through Pgc-1α [46]. At the cellular level, AMPK promotes Pgc-1α activation in a direct or indirect manner: (1) AMPK directly promotes phosphorylating Pgc-1α, the latter interacts with a group of cellular transcription factors including Nrf1/2 and oestrogen related receptor (Errα), activates Tfam, and then initiates the replication of mitochondrial DNA. At the same time, NRFs induce the gene transcription involved in OXPHOS and transfer the protein encoded by nucleus to mitochondria, thus promoting mitochondrial biogenesis [28,46]. (2) AMPK also indirectly regulates Pgc-1α. During exercise or nutritional deficiency, AMPK activation increases the level of intracellular NAD^+^, activates Sirt1 and eventually catalyzes the deacetylation of Pgc-1α [47]. Meanwhile, Sirt1 deacetylates LKB1, and in the subsequent activation of AMPK [48]. Taken together, our results suggest that Cana promotes mitochondrial biogenesis and oxidative capacity by enhancing AMPK and Sirt1 activities and their downstream target, Pgc-1α. Additional studies are needed to confirm these findings.

These studies have several potential limitations. First, Pgc-1α expression has been reported in a variety of tissues, including the skeletal muscle, heart, and brain [7], and the effects of Cana on mitochondrial dynamics in other tissues require further investigation. Second, the effects of Cana on mitochondrial function were only investigated in physiological state, and studies should be performed in rodents or humans with metabolic disorders like insulin resistance and T2DM. Third, mitochondrial oxidative stress and mitophagy are critical for maintaining the mitochondrial homoeostasis [49], and further studies are needed to investigate the effects of Cana on these biological processes.

In conclusion, beyond the well-characterized role in glucose regulation, Cana directly increased cellular energy expenditure of adipocyte by inducing mitochondrial biogenesis and function via an AMPK-Sirt1-Pgc-1α signalling pathway. The present study reveals a new therapeutic function for Cana in regulating energy homoeostasis.

## Supplementary Material

Supplemental Material
