# Supplementary material for: The diabetes medication canagliflozin promotes mitochondrial remodelling of adipocyte via the AMPK-Sirt1-Pgc-1α signalling pathway
Source: Adipocyte. 2020 Aug 23;9(1):484–94. doi: 10.1080/21623945.2020.1807850 (PMC7469612; doi:10.1080/21623945.2020.1807850)
Supplement: Supplemental Material [file KADI_A_1807850_SM4176.docx]

**Supplementary materials:**

**Table S1. List of specific primers used for real time PCR gene expression**

| **Gene** | **Fwd** | **Rev** |
| --- | --- | --- |
| **mSlc5a1** | 5'-ATGCGGCTGACATCTCAGTC-3' | 5'-ACCAAGGCGTTCCATTCAAAG-3' |
| **mSlc5a2** | 5'-ATGGAGCAACACGTAGAGGC-3' | 5'-ATGACCAGCAGGAAATAGGCA-3' |
| **mPgc1α** | 5'-GCACCAGAAAACAGCTCCAAG-3' | 5'-CGTCAAACACAGCTTGACAGC-3' |
| **mPgc1β** | 5'-TCCTGTAAAAGCCCGGAGTAT-3' | 5'-GCTCTGGTAGGGGCAGTGA-3' |
| **mNrf1** | 5'-AGCACGGAGTGACCCAAAC-3' | 5'-TGTACGTGGCTACATGGACCT-3' |
| **mErrα** | 5'-GACGGCAGAAGTACAAACGG-3' | 5'-CAACCAGCAGATGCGACAC-3' |
| **mTfam** | 5'-ATTCCGAAGTGTTTTTCCAGCA-3' | 5'-TCTGAAAGTTTTGCATCTGGGT-3' |
| **mAtpaseβ** | 5'-GGTTCATCCTGCCAGAGACTA-3' | 5'-AATCCCTCATCGAACTGGACG-3' |
| **mCox4β** | 5'-CTGCCCGGAGTCTGGTAATG-3' | 5'-CAGTCAACGTAGGGGGTCATC-3' |
| **mUqcrc2** | 5'-AAAGTTGCCCCGAAGGTTAAA-3' | 5'-GAGCATAGTTTTCCAGAGAAGCA-3' |
| **mUqcrfs1** | 5'-GGTAACTGCAACTACTACTGTGG-3' | 5'-CTTGATCTCGATCTTCGACATGG-3' |
| **mAtp5a1** | 5'-TCTCCATGCCTCTAACACTCG-3' | 5'-CCAGGTCAACAGACGTGTCAG-3' |
| **mMtco2** | 5'-GGATTTGTTCACTGATTCCCATTA-3' | 5'-GCATCTGGGTAGTCTGAGTAGCG-3' |
| **mSDHB** | 5'-AATTTGCCATTTACCGATGGGA-3' | 5'-AGCATCCAACACCATAGGTCC-3' |
| **mNdufb8** | 5'-TGTTGCCGGGGTCATATCCTA-3' | 5'-AGCATCGGGTAGTCGCCATA-3' |
| **mPparα** | 5'-TGCAAACTTGGACTTGAACG-3' | 5'-GATCAGCATCCCGTCTTTGT-3' |
| **mCpt2** | 5'-GAGGCATTTGTCAGG-3' | 5'-GGACAGGATGTTGTGG-3' |
| **mMcad** | 5'-TCCTAAAGCTCCTGCTAATAAAGC-3' | 5'-ATCGCTGGCCCATGTTTAAT-3' |
| **mAcox1** | 5'-CCACATATTGACCCCAAGACC-3' | 5'-AGGCATGTAACCCGTAGCAGC-3' |
| **mUcp1** | 5'-TCTCAGCCGGCTTAATGACTG-3' | 5'-GGCTTGCATTCTGACCTTCAC-3' |
| **mPrdm16** | 5'-ACACGCCAGTTCTCCAACCTGT-3' | 5'-TGCTTGTTGAGGGAGGAGGTA-3' |
| **mElvol3** | 5'-GTGTGCTTTGCCATCTACACG-3' | 5'-CTCCCAGTTCAACAACCTTGC-3' |
| **mCidea** | 5'-TCCTATGCTGCACAGATGACG-3' | 5'-TGCTCTTCTGTATCGCCCAGT-3' |
| **mCox8b** | 5'-TGCTGGAACCATGAAGCCAAC-3' | 5'-AGCCAGCCAAAACTCCCACTT-3' |
| **mDio2** | 5'-CATTGATGAGGCTCACCCTTC-3' | 5'-GGTTCCGGTGCTTCTTAACCT-3' |
| **mTmem26** | 5'-AGGGGCTTCCTTAGGGTTTTC-3' | 5'-CCGTCTTGGATGAAGAAGCTG-3' |
| **mHoxa9** | 5'-CCCCGACTTCAGTCCTTGC-3' | 5'-GATGCACGTAGGGGTGGTG-3' |
| **mSirt1** | 5'-GCTGACGACTTCGACGACG-3' | 5'-TCGGTCAACAGGAGGTTGTCT-3' |
| **mPpar γ** | 5'-GCCCTTTGGTGACTTTATGGA-3' | 5'-GCAGCAGGTTGTCTTGGATG-3' |
| **mC/ebpα** | 5'-CCCACTCAGCTTACAACAGG-3' | 5'-GCTGGCGACATACAGTACAC-3' |
| **mC/ebpβ** | 5'-ACACGTGTAACTGTCAGCCG-3' | 5'-GCTCGAAACGGAAAAGGTTC-3' |
| **mC/ebpδ** | 5'-GGAAGGAACACGGGAAAGC-3' | 5'-AAGTAGAGGCAACGAGGAAT-3' |
| **m18s** | 5'-TTGACTCAACACGGGAAACC-3' | 5'-AGACAAATCGCTCCACCAAC-3' |

**Table S2. List of antibodies used for Western blotting Analysis**

| **Antibody** | **Dilution** | **Catalog** | **Company** |
| --- | --- | --- | --- |
| **Pgc-1α** | 1 : 1000 | Sc-517380 | Santa Cruz, CA, USA |
| **Tfam** | 1 : 1000 | Sc-166965 | Santa Cruz, CA, USA |
| **Cox4** | 1 : 1000 | Sc-517553 | Santa Cruz, CA, USA |
| **Ucp-1** | 1 : 4000 | ab10983 | Abcam, Cambridge, MA, USA |
| **TOMM20** | 1 : 200 | ab186735 | Abcam, Cambridge, MA, USA |
| **Mtco2** | 1 : 1000 | 55070-1-AP | Proteintech, Chicago, IL, USA |
| **Uqcrc2** | 1 : 1000 | ab203832 | Abcam, Cambridge, MA, USA |
| **p-AMPK(Ser 172)** | 1 : 2000 | 2535T | Cell Signaling Technology, MA, USA |
| **AMPK** | 1 : 2000 | 5831T | Cell Signaling Technology, MA, USA |
| **Sirt 1** | 1 : 2000 | [9475](https://www.cellsignal.com/products/primary-antibodies/sirt1-d1d7-rabbit-mab/9475?site-search-type=Products) | Cell Signaling Technology, MA, USA |
| **LKB1** | 1 : 1000 | A2122 | Abclonal Technology, Wuhan, China |
| **β-tubulin** | 1 : 4000 | 200608 | Zen Bio Science Technology, Chengdu, China |
